# Wnt Signaling in Hepatocellular Carcinoma: Biological Mechanisms and Therapeutic Opportunities

**DOI:** 10.3390/cells13231990

**Published:** 2024-12-02

**Authors:** Yingying Zhu, Yajing He, Runliang Gan

**Affiliations:** Key Laboratory of Cancer Cellular and Molecular Pathology in Hunan, Cancer Research Institute, Hengyang Medical School, University of South China, Hengyang 421001, China; 20222013111295@stu.usc.edu.cn (Y.Z.); 20232023111552@stu.usc.edu.cn (Y.H.)

**Keywords:** hepatocellular carcinoma, biological mechanisms, molecular agents, Wnt signaling pathway

## Abstract

Hepatocellular carcinoma (HCC), characterized by significant morbidity and mortality rates, poses a substantial threat to human health. The expression of ligands and receptors within the classical and non-classical Wnt signaling pathways plays an important role in HCC. The Wnt signaling pathway is essential for regulating multiple biological processes in HCC, including proliferation, invasion, migration, tumor microenvironment modulation, epithelial–mesenchymal transition (EMT), stem cell characteristics, and autophagy. Molecular agents that specifically target the Wnt signaling pathway have demonstrated significant potential for the treatment of HCC. However, the precise mechanism by which the Wnt signaling pathway interacts with HCC remains unclear. In this paper, we review the alteration of the Wnt signaling pathway in HCC, the mechanism of Wnt pathway action in HCC, and molecular agents targeting the Wnt pathway. This paper provides a theoretical foundation for identifying molecular agents targeting the Wnt pathway in hepatocellular carcinoma.

## 1. Introduction

Hepatocellular carcinoma (HCC) is currently recognized as the fifth most prevalent malignant neoplasm globally and ranks third in terms of cancer-related mortality. The global incidence of HCC is estimated to be around 500,000 cases annually, with a concerning upward trend in both morbidity and mortality rates over time [1]. The primary causes of HCC include infection with hepatitis B virus (HBV) or hepatitis C virus (HCV), type 2 diabetes mellitus, consumption of aflatoxin-contaminated food, excessive alcohol consumption, obesity, tobacco use, and poor dietary habits [2,3]. Hepatocellular carcinoma development is now understood to be a multi-step process controlled by the accumulation of genetic and epigenetic alterations that inactivate tumor suppressor genes or activate oncogenes, leading to dysregulation of fundamental cellular processes [4]. Several signaling pathways have been implicated in the development of HCC, including the Ras/Raf/MAPK, PI3K/Akt/mTOR, JAK/STAT, Wnt/β-catenin, Hippo, Notch, and Hedgehog pathways, which contribute to HCC by influencing the biological behaviors of hepatocytes, such as proliferation, differentiation, apoptosis, metastasis, and angiogenesis [5].

The Wnt family of proteins comprises cysteine-rich, lipid-modified, secreted glycoproteins. Currently, nearly 20 members of this family have been identified in mammals. Wnt receptors are a group of proteins that serve as ligands for Wnt family proteins. This group includes the Frizzled (Fzd) family of receptors, LDL receptor-related proteins (LRPs) 5 and 6, Ror2, and Ryk. These receptors facilitate distinct intracellular signaling pathways [6]. Wnt signaling pathways encompass both classical and non-classical pathways. In the liver, classical Wnt signaling pathways are particularly important for organ development and maturation. Disruptions in Wnt signaling can lead to hepatic pathogenesis, resulting in various hepatobiliary disorders, both benign and malignant. Moreover, aberrant Wnt signaling contributes to the initiation and progression of diverse liver diseases [7]. This review aims to highlight the significance of the Wnt signaling pathway in HCC development and address the current state of research in this field, hoping to inspire the search for therapeutic approaches in liver cancer.

## 2. Wnt Signaling Pathway

### 2.1. Classical Wnt Pathway

**Figure 1:**
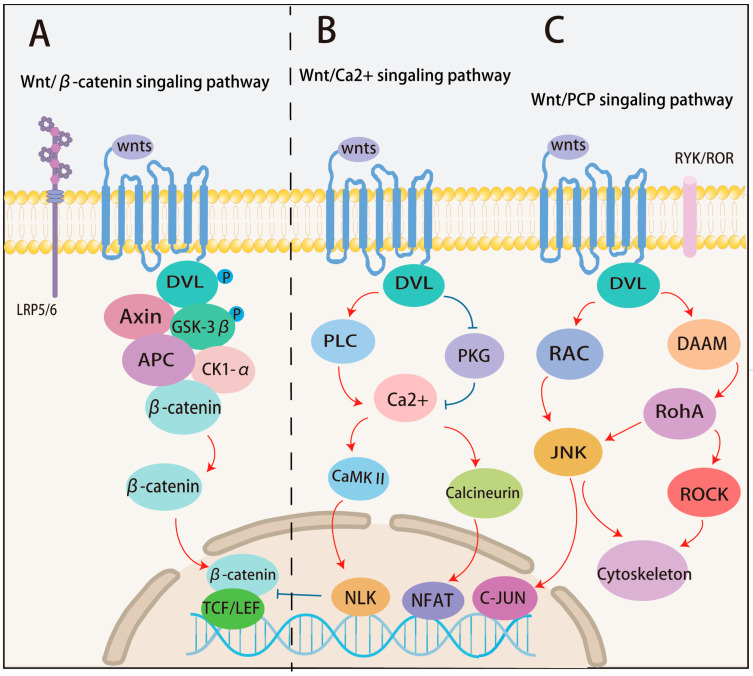
Wnt signaling pathway. (**A**) Classical Wnt signaling pathway. (**B,C**) Non-classical Wnt signaling pathway.

The classical Wnt pathway, known as the Wnt/β-catenin pathway (Figure 1A), is aberrantly activated in liver disease and tumorigenesis. Approximately 20–35% of HCCs exhibit genetic mutations and aberrant activation of key genes involved in this regulatory pathway. Aberrant activation of Wnt/β-catenin, often due to mutations in β-catenin itself, results in accelerated growth and spread of HCC [8]. In the absence of extracellular Wnt ligands, Wnt signaling is maintained in an off state. β-catenin binds to the destruction complex, composed of APC, GSK-3β, Axin, and CK1α. β-catenin is sequentially phosphorylated by CK1α at Ser45 and then by GSK-3β at Ser33, Ser37, and Thr41. Subsequently, it is recognized and ubiquitinated by the E3 ligase subunit βTrCP, leading to β-catenin degradation [9,10]. In the nucleus, TCF/LEF binds to Groucho, forming a repressive complex that inhibits the expression of Wnt target genes [11]. In the presence of extracellular Wnt ligands, which bind to Fzds and activate the classical Wnt pathway, LRP5/6 is phosphorylated by GSK-3β and CK1α, recruiting the scaffolding protein Axin. Fzds recruits Dishevelled (Dvl) to the plasma membrane, which disrupts the destruction complex [12]. In the nucleus, β-catenin acts as a scaffold for TCF/LEF, recruiting co-activators such as CBP, PYGO, LGS/BCL9, and others, leading to the transcription of target genes. Wnt/β-catenin plays an important role in a variety of tumors, promoting proliferation, migration, and invasion in gastric cancer [13], lung cancer [14], colon cancer [15], and other tumors.

### 2.2. Non-Classical Wnt Pathway

Non-classical Wnt pathways primarily include the Wnt-Ca^2+^ and the Wnt-PCP signaling pathways (Figure 1B,C).

#### 2.2.1. Wnt-PCP Signaling Pathway

The Wnt-PCP signaling pathway is typically triggered by Wnt4, Wnt5a, Wnt5b, Wnt7b, and Wnt11 [16,17]. Fzds on the cell membrane surface remain the primary receptors for Wnts, while receptor-like tyrosine kinase (RYK) [18], receptor tyrosine kinase-like orphan receptor 1/2 (ROR1/2) [19], musculoskeletal receptor tyrosine kinase (MUSK) [20], and protein tyrosine kinase 7 (PTK7) [21] serve as secondary receptors for Fzds. The binding of non-classical Wnts to Fzds leads to Dvl phosphorylation and Fzds–Dvl complex formation, and the interaction between Dvl and Dvl-associated activator of morphogenesis (DAAM) activates the small GTPases Rac1 [22] and Ras homologous gene family member A (RhoA) [23]. Rac1 activates JNK [24], which further phosphorylates the c-Jun at Ser63 and Ser73 [25]. RhoA subsequently activates diaphanous1 and Rho-associated coiled-coil containing protein kinase (ROCK) [26]. These PCP effectors lead to lateral asymmetric development of epithelial and other structures, as well as cell polarity and migration by remodeling the cytoskeleton [27]. The Wnt-PCP pathway is upregulated in breast, ovarian, and pancreatic cancers, and its upregulation has been associated with a poor prognosis [28]. Activation of Wnt-PCP signaling enhances self-renewal capacity and sustained migration/invasion of ovarian cancer cells [29]. Vangl2-dependent Wnt-PCP signaling promotes breast cancer cell migration and invasion [30]. Activation of the Wnt-PCP pathway reportedly promotes HCC metastasis [31], although the exact mechanism has not been clarified.

#### 2.2.2. The Wnt-Ca^2+^ Signaling Pathway

The Wnt-Ca^2+^ signaling pathway is primarily triggered by Wnt5a. When Wnts bind to Fzds and Dvl, pertussis toxin-sensitive heterotrimeric G-protein subunits activate phospholipase C (PLC), increasing the cytoplasmic Ca^2+^ level [32]. The increased Ca^2+^ in the concentration activates protein kinase C (PKC) and calmodulin-dependent kinase II (CAMKII). Additionally, the increased calmodulin-dependent phosphatase activity activates the nuclear factor of activated T cells (NFAT) [33]. CAMK II stimulation activates TGFβ-activated kinase 1 (TAK1), followed by nemo-like kinase (NLK) activation, leading to TCF phosphorylation and inhibition of β-catenin/TCF signaling. The Wnt-Fzd-Dvl complex can also inactivate protein kinase G (PKG), which in turn increases Ca^2+^ concentration. The Wnt-Ca^2+^ signaling pathway in colon cancer is associated with an aggressive phenotype in cancer patients, leading to a shorter survival time [34].

## 3. Altered Wnt Signaling in Hepatocellular Carcinoma

### 3.1. Expression of Wnt Ligands in the Classical Pathway in HCC

The activation of classical Wnt pathways is typically initiated by various ligands, such as Wnt1, Wnt2, Wnt3, Wnt3a, Wnt8b, Wnt10a, Wnt10b, and others [35] (Table 1). One possible mode of Wnt secretion involves direct release into the extracellular compartment, where Wnts after endoplasmic reticulum and Golgi modifications move to the cell surface. Wnt molecules then bind via autocrine secretion to receptors on the secreting cells or to neighboring cells via paracrine secretion [36]. Another model involves the transport of Wnts after endoplasmic reticulum and Golgi modification in an endosome-dependent manner. These Wnts are then secreted into the extracellular matrix by exosomes, which are efficient carriers [37]. Wnt1 expression is increased in HCC. Wnt1 binds to the Fzd receptor on target cells, leading to the accumulation and nuclear translocation of β-catenin. In HCC, silencing Wnt1 inhibits HCC cell growth and invasion and induces cell death [38]. LncRNA RPPH1, a competitive endogenous RNA in HCC cells, regulates miR-122 and Wnt1 and their downstream targets, promoting HCC development [39]. Wnt1 activates the β-catenin signaling pathway in M2-type macrophages, leading to the proliferation and malignant transformation of HCC cells. This process is associated with the Wnt-PCP signaling pathway. Cadherin specifically targets and blocks Wnt1 transcription, thereby inactivating the β-catenin signaling cascade in HCC cells [40]. Wnt2b expression is upregulated in HCC and induces pro-tumorigenic effects of HCC tumor-associated macrophages (TAMs) in vivo. This effect is achieved by promoting M2-type macrophage polarization via the Wnt2b/β-catenin signaling pathway. Overexpression of Wnt2b in macrophage cultures significantly increases the expression of EMT-related transcription factors Snail, Twist, and ZEB1 in HCC cells, as well as the mesenchymal markers N-cadherin and vimentin. This effect is attributed to the activation of the Wnt-PCP signaling pathway. In contrast, silencing of Wnt2b or CTNNB1 significantly inhibits the proliferation and migration of HCC cells [41]. Increased expression of Wnt3 in HCC activates the canonical Wnt/β-catenin signaling pathway, resulting in significant transforming activity and morphological changes [42]. Furthermore, it promotes the growth and migration of HCC cells and plays a crucial role in regulating the proliferation of HCC cell lines in vitro [43]. Wnt3 has two N-glycosylation modification sites. The simultaneous mutation of both sites decreases Wnt3’s binding ability to Fzd7, decreasing Wnt-β-catenin protein levels and inhibiting the proliferation, migration, and invasion of HCC [44]. In the context of HCC, Wnt3 triggers the activation of the Wnt/β-catenin signaling pathway by activating Fzd-7 through an autocrine or paracrine mechanism. Wnt3a expression is increased in HCC, and there is a positive correlation between Wnt3a and the expression of Notch3 and Hes1 in the Notch pathway, which synergistically promotes the stem cell-like properties, recurrence, and metastasis of HCC. Suppression of Wnt3a expression inhibits the growth of HCC cells by inducing cell cycle arrest in the G0/G1 phase. It also reduces the expression of c-myc, a gene regulated by Wnt3a, thereby controlling the cell cycle and spread of HCC [45]. The overexpression of Wnt3a is directly associated with the clinical stage, higher tumor–lymph node metastatic stage, and 5-year survival. Additionally, Wnt3a is an independent prognostic factor for HCC that is linked to HBV infection and liver cirrhosis [46]. Following the use of Wnt3 siRNA, the reduction in Wnt3 expression resulted in lower β-catenin levels and a significant reduction in TCF transcriptional activity compared with controls, exhibiting anti-metastatic and anti-proliferative effects [47]. In HCC, Wnt8b expression is elevated and activates the canonical Wnt signaling pathway to promote HCC cell proliferation. ZNF191 stimulates the canonical Wnt/β-catenin signaling pathway, increasing HCC growth. ZNF191, which stimulates the proliferation of liver cancer cells by activating β-catenin and Wnt8b, is linked to the expression of Wnt8b [48,49]. Wnt10b expression is increased in HCC and plays a regulatory role in the cytogenesis, differentiation, and proliferation of HCC by activating the Wnt/β-catenin signaling pathway [50]. A study demonstrated that the knockdown of NSD1, mediated by the CRISPR/Cas9 system, significantly hinders the migration and invasion capacity of HCC cells by deactivating Wnt10b [51].

### 3.2. Expression of Non-Classical Pathway WNT Ligands in HCC

Non-classical Wnt pathways are typically triggered by Wnt5a, Wnt7a, Wnt11, etc. (Table 1). Wnt5a is a tumor suppressor with reduced expression in HCC. Furthermore, there is a negative correlation between the expression of Wnt5a and HCC [52]. Wnt5a overexpression has been reported to inhibit the ability of HCC cells to progress from the G0/G1 phase to the S phase of the cell cycle and reduce their ability to proliferate, invade, and migrate [53]. Moreover, a decrease in the expression of Wnt5a is an independent and reliable indicator of a poor prognosis in patients with HCC [54]. Another study revealed a positive correlation between low levels of cytoplasmic Wnt5a expression and low levels of cytoplasmic Ror2 expression, as well as abnormal β-catenin expression in HCC tissues. Thus, Wnt5a suppresses the biological features of HCC by inhibiting the Ror2-mediated signaling pathway, which hinders the canonical Wnt/β-catenin pathway [55]. In HCC cell lines, knockdown of Wnt5a decreases the production of E-calmodulin, and overexpression of Wnt5a decreases the expression of waveform proteins, demonstrating that Wnt5a can be mediated through EMT-mediated invasion in HCC [56]. Increased expression of Wnt5a reduces the proliferation and EMT promoters Vimentin, SNAI2, MMP2, and MMP9. Consequently, Wnt5a inhibits classical Wnt signaling in HCC cells by suppressing EMT [57]. Expression of Wnt7a is decreased in HCC, and Wnt7a inhibits the migration of HCC cells by reducing the expression of EMT markers. Additionally, overexpression of Wnt7a inhibits the growth and migration of HCC cells and induces apoptosis by decreasing the activity of SKP2/P21 [58]. A study revealed that Wnt11 expression is downregulated in HCC, and overexpression of Wnt11 activates protein kinase C, antagonizes classical Wnt signaling by promoting the phosphorylation of β-catenin, and inhibits proliferation and migration of HCC cells by modulating RhoA/ROCK and Rac1 activities [59].

**Table 1:** Wnt ligands in HCC.

Ligand	Signaling Pathway	Alterations and Roles in HCC	Ref.
Wnt1	Canonical	High expressed in HCC	[38,40]
Wnt2b	Canonical	High expressed in HCC Induced TAMs in HCC	[41]
Wnt3a	Canonical	High expressed in HCC Involved in the regulation of the cell cycle	[45,46]
Wnt8b	Canonical	Activated by ZN191 in HCC	[48,49]
Wnt10b	Canonical	High expressed in HCC	[50]
Wnt5a	Non-canonical	Inhibited EMT, Wnt/β-catenin in HCC	[53,57]
Wnt7a	Non-canonical	Inhibited EMT in HCC	[58]
Wnt11	Non-canonical	Downregulation in HCC	[59]

### 3.3. Altered Expression of Classical Wnt Pathway Receptors

Fzds, 7-transmembrane proteins on the cell membrane surface, act as the primary receptors for Wnts. Additionally, LRPs, single -transmembrane domain proteins, function as secondary receptors for Fzds [60] (Table 2).

Each member of the Fzd family possesses three distinct regions: a seven-transmembrane domain involved in cellular junctions, a C-terminal cytoplasmic domain, and a highly conserved N-terminal extracellular cysteine-rich domain responsible for binding to Wnt ligands. Fzd3 expression is elevated in HCC cells and tissues, while *MDR1*, a target gene of the Wnt pathway, is significantly upregulated in drug-resistant cell lines and HCC tissues. Notably, Fzd3 and *MDR1* expression levels are positively correlated. Knockdown of Fzd3 was found to reverse HCC resistance to cisplatin and adriamycin chemotherapy by inhibiting the Wnt/β-catenin signaling pathway in HCC [61]. Fzd7 is highly expressed in transgenic mouse models of both HCC and human cancers. It reportedly promotes the proliferation and migration of HCC cells through activation of the Wnt/β-catenin signaling pathway. A combined expression analysis of Fzd7 and *GPC3* genes offers higher diagnostic sensitivity and accuracy for early-stage HCC than AFP [62,63]. The Fzd7 receptor binds with Wnt ligands to enhance the durability and initiation of the Wnt/β-catenin signaling pathway. This interaction suppresses the phosphorylation of β-catenin and promotes the growth and motility of HCC cells [64]. Target genes are activated via the Wnt/β-catenin and Hippo signaling pathways when Fzd10 facilitates the nuclear translocation of β-catenin and YAP1. This process promotes tumor growth, HCC stem cell self-renewal, and metastasis. Fzd10 overexpression activates the c-Jun/MEK/ERK axis, rendering HCC cells more resistant to levatinib [65]. LRP5/6 functions as a co-receptor in the Wnt/β-catenin signaling pathway. Upon Wnt binding to Fzd and LRP5/6, GSK-3β activity is decreased, Dvl is activated, and the canonical Wnt pathway is initiated. LRP5/6 expression is significantly elevated in HCC, and its overexpression stimulates HCC development and augments cell proliferation, migration, and invasion [66]. LRP6 exhibits a strong correlation with malignant characteristics and poor prognosis in HCC. It promotes HCC proliferation, invasion, and migration by upregulating Cellular Communication Network Factor 2 (CCN2), which enhances stem cell properties of HCC [67]. BAMBI is a transmembrane glycoprotein that shares significant homology with TGF-β/BMP1-type receptors but lacks the intracellular serine/threonine kinase domain. BAMBI inhibits the canonical Wnt/β-catenin pathway and TGF-β signaling, thereby suppressing HCC development and being associated with a negative prognosis [68].

### 3.4. Alterations in Non-Classical Wnt Signaling Pathway Receptors

Fzd2 expression is upregulated in HCC tissues. This upregulation promotes cell proliferation, migration, and invasion; inhibits apoptosis; and enhances of the stem cell properties of HCC [69]. Fzd2 can induce EMT via the non-canonical Wnt pathway, and suppressing Fzd2 decreases the invasive and migratory capabilities of HCC cells. Phosphorylation of the EMT-inducing factor Stat3, regulated by the Wnt5/Fzd2 signaling pathway in HCC cell lines, is associated with Fzd2-dependent EMT and cell migration, contributing to the occurrence of EMT [70]. Ror2, a member of the tyrosine kinase superfamily, acts as a co-receptor for receptor complexes alongside the Fzd receptor. Its primary function is to serve as a receptor for Wnt5a in the non-canonical Wnt pathway [71]. The Wnt5a/Ror2 signaling pathway is involved in HCC differentiation. Decreased Ror2 expression activates the non-canonical Wnt pathway, which stimulates the canonical Wnt pathway. This activation leads to the dedifferentiation of HCC cells, a prognostic indicator for HCC [72].

**Table 2:** Wnt receptors and co-receptors in HCC.

Receptor	Signaling Pathway	Alterations and Roles in HCC	Ref.
Fzd3	Canonical	Highly expressed in HCC	[61]
Fzd7	Canonical	Highly expressed in HCC	[62,63]
Fzd10	Canonical	Highly expressed in HCC	[65]
LRP6	Canonical	Activated by stemness in HCC	[67]
Bambi	Canonical	Downregulation in HCC	[68]
Fzd2	Non-canonical	Highly expressed in HCC Driver of EMT in HCC	[69,70]
Ror2	Non-canonical	Downregulation in HCC	[72]

## 4. Wnt Signaling Regulates the Biological Behavior of Hepatocellular Carcinoma Cells

### 4.1. Regulation of HCC Cell Proliferation, Migration, and Apoptosis

**Figure 2:**
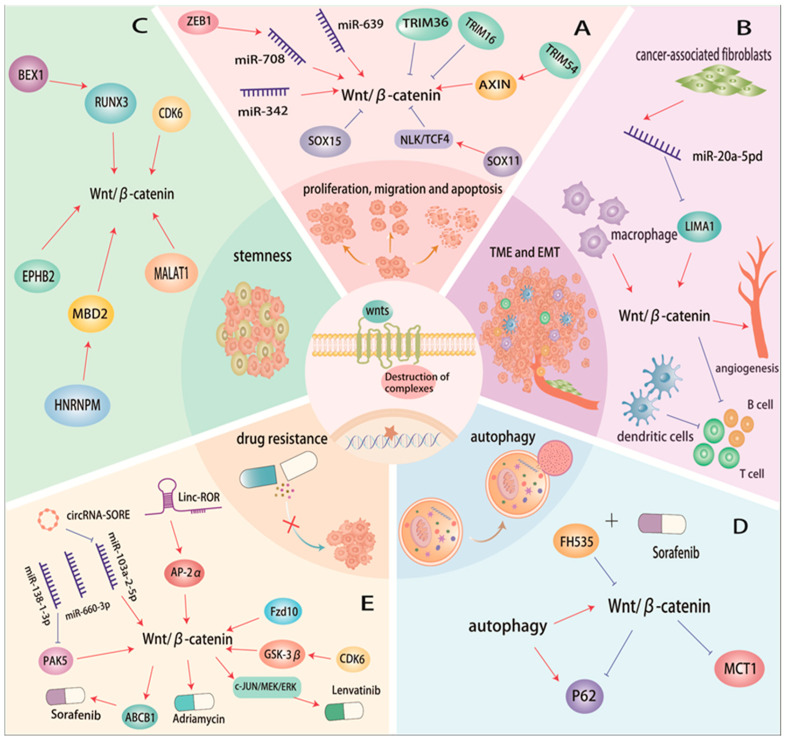
Wnt signaling regulates HCC biological behavior. (**A**) Biomolecules act on Wnt signaling pathway to regulate proliferation, invasion, and apoptosis in HCC. (**B**) Tumor microenvironment acts on Wnt signaling pathway to regulate HCC. (**C**) Wnt signaling pathway regulates stem cell properties to regulate HCC. (**D**) Dual role of autophagy in hepatocellular carcinoma is achieved through Wnt pathway. (**E**) Wnt signaling pathway plays a role in drug resistance.

The Wnt signaling system regulates the growth, migration, and survival of HCC cells (Figure 2A). Zinc finger E box-binding homeobox 1 (ZEB1) stimulates the Wnt/β-catenin signaling cascade, which is regulated by miR-708, in hepatocellular carcinoma cells, enhancing their proliferation, motility, and invasion and suppressing cell death [73]. Furthermore, miR-342 and miR-639 can regulate the growth and apoptosis of hepatocellular carcinoma cells by influencing the Wnt/β-catenin signaling pathway [74,75]. Tripartite-motif (TRIM) proteins are a specific group of E3 ubiquitin ligases. Alterations in TRIM expression have been linked to cancer metastasis and poor prognosis in HCC patients. TRIM54 is upregulated in HCC and activates the Wnt/β-catenin signaling pathway by ubiquitinating Axin1. This activation stimulates HCC cell growth and migration. Additionally, increased TRIM54 expression is associated with HCC occurrence and poor prognosis [76]. Conversely, TRIM16 promotes β-catenin ubiquitination, suppressing the Wnt/β-catenin signaling pathway in HCC. Consequently, decreased β-catenin levels inhibit the proliferation, motility, and invasion of HCC cells [77]. TRIM36 facilitates apoptosis by interacting with Caspase-3 and Caspase-7, apoptotic effectors. Moreover, TRIM36 enhances apoptosis and inhibits the growth, migration, and invasion of HCC cells by suppressing the Wnt/β-catenin signaling pathway [78]. SRY-Box transcription factor (SOX) is a member of the transcription factor (TF) family, while NLK functions as a MAPK-like kinase regulating oncogenic signaling pathways [79]. In HCC tissues, SOX11 increased TCF4 phosphorylation and NLK expression, inhibiting the Wnt/β-catenin signaling pathway. As a result, apoptosis was induced, and HCC cell proliferation was suppressed [80,81]. A favorable prognosis for HCC is associated with decreased Sox15 expression, which also deactivates the Wnt pathway to inhibit HCC growth. Furthermore, hypermethylation of the SOX15 promoter inhibits HCC development and proliferation by blocking the Wnt/β-catenin signaling pathway [82].

### 4.2. Tumor Microenvironment and EMT

The tumor microenvironment (TME) comprises immune cells, blood vessels, extracellular matrix (ECM), fibroblasts, lymphocytes, bone marrow-derived inflammatory cells, and signaling factors [83]. Wnt ligands, secreted by stromal and inflammatory cells within the extracellular matrix of the TME, facilitate tumor invasion, migration, and immune tolerance (Figure 2B). The TME stimulates EMT in tumor cells, promoting tumor metastasis. EMT, which promotes the survival of cancer stem cells (CSCs), involves WNT signaling [84]. Within the TME, the presence of CSCs is linked to HCC aggressiveness, drug resistance, and recurrence [85].

Cancer-associated fibroblasts (CAFs) are essential components of the TME, supporting and regulating various tumor cell processes, including angiogenesis, migration, invasion, apoptosis, proliferation, and drug resistance [86]. Tumor cells undergoing EMT are recognized as one of the sources of CAFs in the TME [87]. The tumor suppressor gene *LIMA1* inhibits HCC progression by suppressing the Wnt/β-catenin signaling pathway. CAFs secrete exosomes to regulate tumor progression, and their exosomal miR-20a-5p mediates the downregulation of *LIMA1* levels and promotes the malignant phenotype of HCC [88]. EMT is strongly associated with not only invasion and metastasis but also proliferation [89]. EMT is crucial during the early stages of HCC cell invasion and metastasis [90]. In HCC, the activation of classical Wnt signaling triggers the interaction between intracellular E-calmodulin and β-catenin, forming a complex. This complex induces EMT, facilitating HCC cell invasion and metastasis [91]. TDP-43, highly expressed in HCC, activates the Wnt/β-catenin signaling pathway by inhibiting GSK-3β protein translation and suppressing β-catenin phosphorylation. This, in turn, activates downstream target genes *CyclinD1* and *c-Myc*, induces EMT, and promotes HCC progression [92].

TAMs are a significant component of the TME. TAMs contribute to tumor progression, angiogenesis, metastasis, and immune suppression by producing various cytokines, chemokines, growth factors, and matrix metalloproteinases (MMPs). Wnt ligands secreted by macrophages can activate the Wnt signaling pathway in cancers cells. Cancer cells can also activate the Wnt/β-catenin signaling pathway in macrophages by secreting Wnt ligands. Through paracrine mechanisms, HCC cells can activate the Wnt/β-catenin signaling pathway, leading to M2 macrophage polarization and promoting tumor growth [93,94]. Dendritic cells (DCs) exhibit higher levels of β-catenin and GSK-3β, two molecules involved in the Wnt signaling pathway, than monocytes. The crosstalk between Wnt and MAPK signaling pathways has been implicated in dendritic cell differentiation, maturation, and function [95]. β-catenin activation contributes to immune evasion in HCC by affecting DC recruitment and antigen-specific T cell function [96].

HCC patients harboring CTNNB1 mutations exhibit a significant decrease in the abundance of CD8+ T cells, CD4+ T cells, Th2 cells, Tfh cells, and B cells [97]. Moreover, the Wnt/β-catenin signaling pathway is essential for regulating angiogenic factors, stimulating tumor angiogenesis. HCC is characterized by hypervascularity and vascular abnormalities, and Wnt signaling is necessary for angiogenesis [98].

### 4.3. Stemness

Significant CSC features are present in HCC, and growing evidence indicates that tumor stem cells and immune evasion are crucial factors in tumor development, progression, and metastasis (Figure 2C). BEX1, an HCC stem cell marker, promotes HCC tumorigenesis and stemness. BEX1 interacts with RUNX3 to inhibit RUNX3-mediated β-catenin transcriptional repression, activating the Wnt/β-catenin signaling pathway and maintaining CSC-HCC stemness. Downregulation of BEX1 expression releases RUNX3 and represses Wnt/β-catenin signaling in non-CSC-HCC [99]. The overexpression of Metastasis-associated lung adenocarcinoma transcript 1 (MALAT1), a transcriptional regulator, is observed in HCC and is considered a biomarker for poorly differentiated HCC cells. MALAT1 expression is regulated through its interaction with the Wnt/β-catenin signaling pathway. The interaction between MALAT1 expression and the Wnt/β-catenin signaling pathway regulates HCC oncogenicity and stem cell properties. Suppression of MALAT1 decreases β-catenin levels and inhibits the Wnt/β-catenin signaling pathway, hindering HCC stemness maintenance, growth, and metastasis [100]. Upregulation of HNRNPM in HCC cells significantly contributes to the acquisition of CSC characteristics and tumorigenesis in HCC while also suppressing apoptosis. *HNRNPM* promotes selective splicing of MBD2. Additionally, MBD2a and MBD2c isoforms can activate HNRNPM expression in HCC. Inhibition of HNRNPM impedes HCC progression and enhances the efficacy of Wnt-activated HCC treatment with PD 1 blocking immunotherapy [101]. EPHB2 is a member of the EPH receptor family, comprising transmembrane glycoproteins with receptor tyrosine kinase activity. Increased EPHB2 expression activates the Wnt/β-catenin signaling pathway, enhancing HCC stemness, regulating HCC growth, and increasing resistance to sorafenib [102].

### 4.4. Autophagy

Autophagy has a dual role in the growth and progression of cancer. On one hand, it can function as a tumor suppressor by eliminating oncogenic proteins, inducing cellular apoptosis, triggering stress and immune responses, inhibiting reactive oxygen species, suppressing inflammatory processes, and maintaining genomic integrity [103]. On the other hand, autophagy can promote tumor growth by providing energy and nutrients, reducing oxidative stress and hypoxia, and stimulating angiogenesis [104]. 2,5-Dichloro-N-(2-methyl-4-nitrophenyl) benzenesulfonamide (FH535) is a synthetic compound that inhibits the Wnt/β-catenin pathway. It exhibits anti-proliferative and anti-angiogenic effects on various cancer cell types [105]. A link exists between autophagy and the Wnt/β-catenin signaling pathway, where β-catenin acts as a transcriptional regulator of the autophagy marker p62 in the canonical Wnt pathway [106,107]. Upon FH535 treatment, p62mRNA levels increase. FH535 and its derivative FH535-N efficiently inhibit HCC cell proliferation and induce apoptosis by suppressing β-catenin’s transcriptional activity. Moreover, the combination of FH535 and sorafenib enhances the effects on HCC cell proliferation, apoptosis, and autophagy [108]. Autophagy acts as a protective mechanism for HCC cells under hypoxic and nutrient-deprived conditions. HCC cells rely on autophagy for survival and proliferation as it modulates lipid metabolism in hypoxic environments [109]. Monocarboxylate transporter protein 1 (MCT1) plays a crucial role in facilitating lactate transport and H+ clearance in cancer cells [110]. MCT1 is significantly upregulated in HCC tissues. Autophagy enhances HCC cells invasion and migration by activating the Wnt/β-catenin signaling pathway, which regulates glycolysis in HCC cells. Downregulation of β-catenin inhibits autophagy-induced glycolysis and decreases MCT1 expression in HCC cells [111].

### 4.5. Drug Resistance

The emergence of drug resistance in HCC patients diminishes the efficacy of drug-based HCC treatments. Notably, the Wnt signaling pathway significantly contributes to the development of drug resistance in HCC (Figure 2E). Lenvatinib, a potent inhibitor of multiple receptor tyrosine kinases (RTKs), is a primary targeted therapeutic option for advanced HCC. Activation of the β-catenin/c-JUN/MEK/ERK pathway through the receptor Fzd10 in the canonical Wnt pathway is crucial for the development of lenvatinib resistance. Targeting Fzd10 can restore sensitivity to lenvatinib in HCC [65]. Cell cycle protein-dependent kinase 6 (CDK6) binds to and regulates GSK3β activity. Through this interaction, HCC cells’ stemness properties are enhanced, and the Wnt/β-catenin signaling pathway is stimulated. HCC patients resistant to lenvatinib exhibit significantly higher levels of CDK6 expression compared with responsive patients. Reprogramming of CDK6 kinase is a critical factor in the development of lenvatinib resistance. Targeting CDK6 has been shown to improve lenvatinib resistance in HCC [112].

Sorafenib is a potent inhibitor of multiple kinases, and the presence of N6-methyladenosine-modified circRNA-SORE has been found to contribute to the development of sorafenib resistance in HCC. In HCC, circRNA-SORE increases resistance to sorafenib treatment by acting as a competitive activator of the Wnt/β-catenin pathway. It sponges both miR-660-3p and miR-103a-2-5p, competitively activating the Wnt/β-catenin pathway. This activation ultimately leads to sorafenib resistance in HCC [113]. P21-Activated Kinases 5 (PAK5) is a highly conserved serine/threonine protein kinase. Research has shown that miR-138-1-3p enhances the susceptibility of HCC cells to sorafenib by selectively targeting PAK5. In vitro experiments have demonstrated that PAK5 reduces sorafenib-induced apoptosis in HCC cells. PAK5 promotes the transcriptional activation of ABCB1 through the Wnt/β-catenin pathway. PAK5 facilitates ABCB1 transcriptional activation and nuclear translocation of β-catenin. Moreover, β-catenin binding to the ABCB1 promoter and activating its transcriptional activation contribute to sorafenib resistance [114].

Adriamycin is a commonly used chemotherapeutic agent for advanced HCC. Linc-ROR, a long intergenic non-coding RNA, is upregulated in adriamycin-resistant HCC cells, suggesting its oncogenic role. A study investigated the role of Linc-ROR in modulating adriamycin sensitivity and its impact on cell proliferation, EMT, and metastasis in vitro. Suppressing Linc-ROR could inhibit EMT and increases the sensitivity of HCC cells to adriamycin, both in vitro and in vivo. The study findings suggested that Linc-ROR contributes to Wnt/β-catenin signaling activation in HCC, promoting carcinogenesis and adriamycin resistance, an effect mediated through the Linc-ROR/AP-2α/Wnt/β-catenin axis [115].

## 5. Molecular Agents Targeting the Wnt Pathway in Hepatocellular Carcinoma

### 5.1. Drugs Targeting Cell Membrane Molecules

The Secreted Frizzled-Related Proteins (SFRPs) genes specifically encode the extracellular structural domain of Fzd and function as natural inhibitors of the Wnt/β-catenin pathway. They achieve this by binding to the Wnt ligand and blocking its interaction with Fzd. The expression of SFRP3, a well-known inhibitor of the Wnt signaling pathway, is reduced in HCC tissues. When overexpressed, SFRP3 inhibits the Wnt/β-catenin signaling pathway, preventing HCC growth, migration, and invasion [116]. SFRP1 acts as a suppressor of the Wnt signaling pathway. Deletion or downregulation of SFRP1 expression significantly impacts HCC. SFRP1 activates the Wnt/β-catenin pathway by enhancing the binding of Wnt16B to its receptor FZD7, leading to increased β-catenin activity [117]. Vantictumab (OMP-18R5) can interact with the Fzd receptor to block Wnt signaling [118]. In a Phase I clinical trial (NCT02069145), the Fz8-Fc fusion protein Ipafricept (OMP-54F28) inhibited the competitive binding of Wnt ligands to Fzd8 and could be used in combination with sorafenib in patients with advanced HCC [119].

The utilization of Soluble frizzled-7 (Sfzd7) peptides can selectively nullify the functional efficacy of Fzd7 in HCC. Sfzd7 peptides have been shown to negatively impact both cell proliferation and the Wnt/β-catenin signaling pathway. These peptides have the potential to be widely used in HCC treatment, especially in combination with conventional chemotherapies or drugs targeting other components of the Wnt/β-catenin signaling pathway. These peptides can modulate the Wnt/β-catenin signaling pathway in HCC, thereby enhancing cellular sensitivity to therapeutic agents [120]. Dickkopf-1 (DKK1) expression is elevated in HCC [121], promotes β-catenin expression, and upregulates the expression of RhoA and JNK of the non-classical Wnt pathway to promote invasion and metastasis in HCC [122]. DKN-01, a humanized monoclonal antibody targeting DKK1, binds to the co-receptors LRP5/6 and inhibits Wnt signaling. A clinical trial (NCT03645980) investigated the effects of DKN-01 alone and in combination with sorafenib in patients with advanced HCC [123]. S-allylmercapto cysteine (SAMC), a water-soluble active compound extracted from Chenopodium album, acts as an anti-tumor agent in HCC by directly targeting LRP6 on HCC cell membranes [124].

### 5.2. Drugs Targeting Cytoplasmic Molecules

Tankyrase inhibitors, including Tankyrase 1 (TNKS1) and Tankyrase 2 (TNKS2), are involved in various cellular processes. These enzymes are responsible for the degradation of AXIN, promoting β-catenin activity. In HCC, TNKS1 and TNKS2 expression is upregulated. Tankyrase inhibitors, such as XAV939 and its derivative WXL-8, stabilize AXIN1 and AXIN2 proteins in HCC cell lines. These inhibitors reduce AXIN2 protein levels, regulating the Wnt/β-catenin pathway and reducing β-catenin/TCF4 transcriptional activity [125,126]. CK1 inhibitors, such as pyrvinium, bind to CK1α, activating and upregulating GSK3β expression. This leads to β-catenin phosphorylation and subsequent inhibition of the Wnt/β-catenin signaling pathway [127]. PORCN inhibitors are Membrane-bound O-acyltransferases (MBOATs) that acylate Wnt ligands in the endoplasmic reticulum. Their primary function is to facilitate Wnt ligand secretion by attaching palmitoyl groups to Wnt proteins [128]. It was demonstrated that LGK974 selectively inhibits PORCN, blocking Wnt signaling to inhibit tumor growth [129]. CGX1321, a novel PORCN inhibitor for clinical trials in solid tumors, has been tested in a Phase I clinical trial in HCC patients (NCT03507998) [130]. Regarding β-catenin inhibitors, the FDA-approved psychiatric drug pimozide (PMZ) has been reported to inhibit HCC proliferation by disrupting the classical Wnt pathway, decreasing downstream expression of EpCAM and decreasing β-catenin expression [131]. PRI-724 inhibits the Wnt signaling pathway by interfering with the interaction between β-catenin and CREB binding protein (CBP) [132]. PRI-724 exerts antitumor effects on constitutive β-catenin-activated hepatocellular carcinoma cell lines by decreasing cell proliferative activity and enhancing apoptosis [133]. GATA5 inhibits β-catenin expression and interacts with it to prevent nuclear entry, thereby reducing the expression of downstream genes and suppressing HCC development [134].

### 5.3. Drugs Targeting the Nucleus

**Figure 3:**
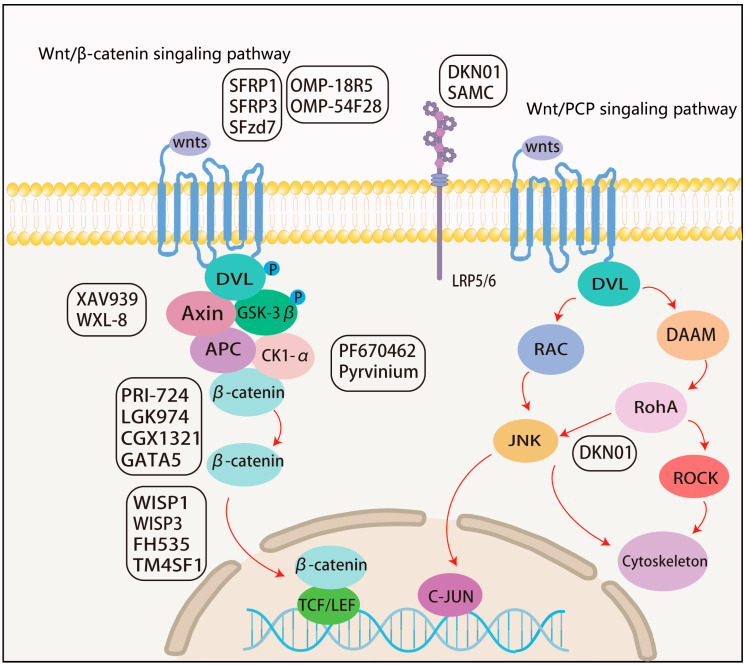
Molecular drugs and targets targeting the Wnt pathway in hepatocellular carcinoma.

*FH535* targets the Wnt/β-catenin signaling pathway, preventing the binding of β-catenin coactivators to target genes and inhibiting Wnt signaling [135]. *FH535* has been shown to restore sensitivity to lenvatinib in HCC cell lines or primary HCC cells that overexpress Fzd10. Additionally, FH535 promotes the characterization of CSC differentiation and regulates β-catenin activity, inhibiting downstream signaling pathways and preventing HCC development [136]. Expression of Wnt1-induced signaling pathway protein 1 (WISP1), a downstream target gene of β-catenin, is positively associated with HCC development [137]. Wnt1-induced signaling pathway protein 3 (WISP3) is downregulated in HCC and inversely associated with HCC development. WISP3 inhibits β-catenin nuclear translocation by activating GSK-3β, suppressing β-catenin/TCF/LEF signaling and impeding HCC progression [138]. Increased TM4SF1 protein expression in HCC activates the Wnt/β-catenin signaling pathway and upregulated target genes Axin2 and cyclin1. Thus, TM4SF1 is a promising therapeutic target for HCC treatment of HCC [139] (Figure 3) (Table 3).

## 6. Discussion

The Wnt signaling pathway is a conserved and regulated molecular mechanism, and its dysregulation plays a role in various malignancies. Several components of the canonical Wnt pathway significantly influence the development of HCC. Wnt signaling in HCC can target β-catenin, an essential component of the canonical Wnt signaling pathway. The Wnt/PCP pathway and the Wnt-Ca^2+^ pathway, non-canonical Wnt signaling pathways, can regulate HCC cell development, invasion, and motility by inducing EMT. Furthermore, they can influence the canonical Wnt pathway, which can impact HCC development.

Aberrant regulation of the Wnt signaling pathway is associated with the aberrant transcription of several oncogenes. Wnt ligands are highly expressed in HCC and promote the canonical Wnt/β-catenin pathway, contributing to HCC growth and progression. They are key players in the classical Wnt signaling pathway. Conversely, reduced expression of Wnt ligands in the non-canonical Wnt pathway inhibits the development and progression of HCC. Studies have demonstrated that biomolecules such as ZEB1, TRIM, and SOX can regulate cell proliferation and apoptosis in HCC by influencing the Wnt/β-catenin pathway. By activating EMT, the Wnt signaling pathway within the tumor microenvironment facilitates HCC invasion and migration. Furthermore, TAMs in the tumor microenvironment activate the Wnt/β-catenin pathway, which aids HCC cells angiogenesis and immune evasion. BEX1, MALAT1, and HNRNPM, among others, can modulate HCC through the Wnt pathway, thereby regulating HCC stem cell characteristics and subsequently influencing HCC proliferation, migration, and invasion. A relationship exists between the Wnt/β-catenin pathway and autophagy. The Wnt/β-catenin pathway inhibits autophagy in HCC, resulting in enhanced HCC cell proliferation and decreased cell apoptosis. Lenvatinib, sorafenib, and adriamycin have been utilized to manage advanced HCC. However, the effectiveness of pharmacological therapy is limited by the emergence of resistance. Treatment resistance in HCC has been linked to the activation of the Wnt signaling pathway. Therefore, targeting the Wnt signaling pathway could offer innovative approaches to address drug resistance in HCC.

Growing evidence suggests that the Wnt signaling pathway plays a critical role in HCC. Several molecular medicines targeting the Wnt pathway have shown varying impacts on HCC treatment. Targeted therapy against the Wnt pathway could offer a novel approach to treating HCC and provide hope for HCC patients.

## Figures and Tables

**Table 3:** Molecular agents targeting the Wnt pathway in HCC.

Drug	Mechanisms on Wnt Signaling Pathway	Clinical Trails and Phase	Ref.
SFRP1/SFRP3	Binding to Wnt ligand and inhibiting its interaction with FZD		[116,117]
OMP-18R5	Interacts with Fzd receptors		[118]
OMP-54F28	Inhibition of competitive binding of Wnt ligands to Fzd8	NCT02069145/I	[119]
Sfzd7	Specific elimination of functional activity of Fzd7 in HCC		[120]
DKN-01/DKN-01 United Sorafeni	Binds to the co-receptor LRP5/6	NCT03645980/II NCT03645980/I	[123]
SAMC	Direct targeting of LRP6 on HCC cell membranes		[124]
XAV939/WXL-8	Stabilizes AXIN protein levels and reduces β-catenin transcriptional activity		[125,126]
Pyrvinium	Activation of CK1α leads to beta-linked protein phosphorylation		[127]
LGK974/CGX1321	Selective inhibition of PORCN		[129,130]
PMZ	Decreases β-catenin and EpCAM protein expression.		[131]
PRI-724	Blocking the interaction between β-catenin and CBP		[133]
GATA5	Inhibition of β-catenin expression		[134]
FH535	Inhibition of β-catenin co-activator recruitment to target genes		[136]
WISP1/WISP3	Negative regulation of β-catenin/TCF/LEF signaling		[137,138]
TM4SF1	Upregulation of target genes Axin2 and cyclin1		[139]

Curegenix Inc. & Merck, S. and D.C. Phase I Dose-Escalation Study of CGX-1321 in Subjects with Advanced Gastrointestinal Tumors (NCT03507998) (2018). Available online at: http://clinicaltrials.gov/ct2/show/NCT03507998 (accessed on 4 November 2024). OncoMed Pharmaceuticals, Inc. (Redwood City, CA, USA). A Phase 1b Dose Escalation Study of OMP-54F28 in Combination with Sorafenib in Patients with Hepatocellular Cancer (2014). Available online at: https://clinicaltrials.gov/ct2/show/NCT02069145 (accessed on 4 November 2024). Marquardt, D. J. U. A Phase I/II Multicenter, Open-label Study of DKN-01 to investigate the anti-tumor activity and safety of DKN-01 in patients with hepatocellular carcinoma and Wnt signaling alterations. Available online at: https://clinicaltrials.gov/ct2/show/NCT03645980 (accessed on 4 November 2024).

## Data Availability

Not applicable.

## Abbreviations

HCC	Hepatocellular carcinoma
EMT	Epithelial–mesenchymal transition
HBV	Hepatitis B virus
HCV	Hepatitis C virus
Fzd	Frizzled
LRPs	LDL receptor-related proteins
Dvl	Dishevelled
RYK	Receptor-like tyrosine kinase
ROR1/2	Receptor tyrosine kinase-like orphan receptor 1/2
MUSK	musculoskeletal receptor tyrosine kinase
PTK7	Protein tyrosine kinase 7
DAAM	Dvl-associated activator of morphogenesis
RhoA	Ras homologous gene family member A
ROCK	Rho-associated coiled-coil containing protein kinase
PLC	Phospholipase C
PKC	Protein kinase C
CAMKII	Calmodulin-dependent kinase II
NFAT	Nuclear factor of activated T cells
TAK1	TGFβ-activated kinase 1
NLK	Nemo-like kinase
PKG	Protein kinase G
TAMs	Tumor-associated macrophages
ZEB1	Zinc finger E box-binding homeobox 1
TRIM	Tripartite-motif
SOX	SRY-Box
TF	Transcription factor
TME	Tumor microenvironment
ECM	Extracellular matrix
CSCs	Cancer stem cells
CAFs	Cancer-associated fibroblasts
MMPs	Matrix metalloproteinases
DCs	Dendritic cells
MALAT1	Metastasis-associated lung adenocarcinoma transcript 1
FH535	2,5-Dichloro-N-(2-methyl-4-nitrophenyl) benzenesulfonamide
MCT1	Monocarboxylate transporter protein 1
RTKs	Receptor tyrosine kinases
CDK6	Cell cycle protein-dependent kinase 6
PAK5	P21-Activated Kinases 5
SFRPs	Secreted Frizzled-Related Proteins
Sfzd7	Soluble frizzled-7
DKK1	Dickkopf-1
SAMC	S-allylmercapto cysteine
TNKS1	Tankyrase 1
TNKS2	Tankyrase 2
MBOATs	Membrane-bound O-acyltransferase
PMZ	Pimozide
CBP	CREB binding protein
WISP1	Wnt1-induced signaling pathway protein 1
WISP3	Wnt1-induced signaling pathway protein 3

## References

[1] Sung H., Ferlay J., Siegel R.L., Laversanne M., Soerjomataram I., Jemal A., Bray F. (2021). Global Cancer Statistics 2020: GLOBOCAN Estimates of Incidence and Mortality Worldwide for 36 Cancers in 185 Countries. CA A Cancer J. Clin..

[2] Baecker A., Liu X., La Vecchia C., Zhang Z.F. (2018). Worldwide incidence of hepatocellular carcinoma cases attributable to major risk factors. Eur. J. Cancer Prev..

[3] Llovet J.M., Kelley R.K., Villanueva A., Singal A.G., Pikarsky E., Roayaie S., Lencioni R., Koike K., Zucman-Rossi J., Finn R.S. (2021). Hepatocellular carcinoma. Nat. Rev. Dis. Primers.

[4] Wang Y., Deng B. (2023). Hepatocellular carcinoma: Molecular mechanism, targeted therapy, and biomarkers. Cancer Metastasis Rev..

[5] Garcia-Lezana T., Lopez-Canovas J.L., Villanueva A. (2021). Signaling pathways in hepatocellular carcinoma. Adv. Cancer Res..

[6] Hayat R., Manzoor M., Hussain A. (2022). Wnt signaling pathway: A comprehensive review. Cell Biol. Int..

[7] Perugorria M.J., Olaizola P., Labiano I., Esparza-Baquer A., Marzioni M., Marin J.J.G., Bujanda L., Banales J.M. (2019). Wnt-β-catenin signalling in liver development, health and disease. Nat. Rev. Gastroenterol. Hepatol..

[8] Deldar Abad Paskeh M., Mirzaei S., Ashrafizadeh M., Zarrabi A., Sethi G. (2021). Wnt/β-Catenin Signaling as a Driver of Hepatocellular Carcinoma Progression: An Emphasis on Molecular Pathways. J. Hepatocell. Carcinoma.

[9] Stamos J.L., Weis W.I. (2013). The β-catenin destruction complex. Cold Spring Harb. Perspect. Biol..

[10] Wu G., Xu G., Schulman B.A., Jeffrey P.D., Harper J.W., Pavletich N.P. (2003). Structure of a beta-TrCP1-Skp1-beta-catenin complex: Destruction motif binding and lysine specificity of the SCF(beta-TrCP1) ubiquitin ligase. Mol. Cell.

[11] Staal F.J., Luis T.C., Tiemessen M.M. (2008). WNT signalling in the immune system: WNT is spreading its wings. Nat. Rev. Immunol..

[12] Murillo-Garzón V., Kypta R. (2017). WNT signalling in prostate cancer. Nat. Rev. Urol..

[13] Wu Q., Ma J., Wei J., Meng W., Wang Y., Shi M. (2021). lncRNA SNHG11 Promotes Gastric Cancer Progression by Activating the Wnt/β-Catenin Pathway and Oncogenic Autophagy. Mol. Ther..

[14] Pan J., Fang S., Tian H., Zhou C., Zhao X., Tian H., He J., Shen W., Meng X., Jin X. (2020). lncRNA JPX/miR-33a-5p/Twist1 axis regulates tumorigenesis and metastasis of lung cancer by activating Wnt/β-catenin signaling. Mol. Cancer.

[15] Zhao H., Ming T., Tang S., Ren S., Yang H., Liu M., Tao Q., Xu H. (2022). Wnt signaling in colorectal cancer: Pathogenic role and therapeutic target. Mol. Cancer.

[16] Yang Y., Mlodzik M. (2015). Wnt-Frizzled/planar cell polarity signaling: Cellular orientation by facing the wind (Wnt). Annu. Rev. Cell Dev. Biol..

[17] Butler M.T., Wallingford J.B. (2017). Planar cell polarity in development and disease. Nat. Rev. Mol. Cell Biol..

[18] Fradkin L.G., Dura J.M., Noordermeer J.N. (2010). Ryks: New partners for Wnts in the developing and regenerating nervous system. Trends Neurosci..

[19] Katoh M. (2005). WNT/PCP signaling pathway and human cancer (review). Oncol. Rep..

[20] Gordon L.R., Gribble K.D., Syrett C.M., Granato M. (2012). Initiation of synapse formation by Wnt-induced MuSK endocytosis. Development.

[21] Han J.D., Bertin N., Hao T., Goldberg D.S., Berriz G.F., Zhang L.V., Dupuy D., Walhout A.J., Cusick M.E., Roth F.P. (2004). Evidence for dynamically organized modularity in the yeast protein-protein interaction network. Nature.

[22] Gombos R., Migh E., Antal O., Mukherjee A., Jenny A., Mihály J. (2015). The Formin DAAM Functions as Molecular Effector of the Planar Cell Polarity Pathway during Axonal Development in Drosophila. J. Neurosci..

[23] Aspenström P., Richnau N., Johansson A.S. (2006). The diaphanous-related formin DAAM1 collaborates with the Rho GTPases RhoA and Cdc42, CIP4 and Src in regulating cell morphogenesis and actin dynamics. Exp. Cell Res..

[24] Coso O.A., Chiariello M., Yu J.C., Teramoto H., Crespo P., Xu N., Miki T., Gutkind J.S. (1995). The small GTP-binding proteins Rac1 and Cdc42 regulate the activity of the JNK/SAPK signaling pathway. Cell.

[25] Dérijard B., Hibi M., Wu I.H., Barrett T., Su B., Deng T., Karin M., Davis R.J. (1994). JNK1: A protein kinase stimulated by UV light and Ha-Ras that binds and phosphorylates the c-Jun activation domain. Cell.

[26] Kitzing T.M., Sahadevan A.S., Brandt D.T., Knieling H., Hannemann S., Fackler O.T., Grosshans J., Grosse R. (2007). Positive feedback between Dia1, LARG, and RhoA regulates cell morphology and invasion. Genes. Dev..

[27] VanderVorst K., Dreyer C.A., Konopelski S.E., Lee H., Ho H.H., Carraway K.L. (2019). Wnt/PCP Signaling Contribution to Carcinoma Collective Cell Migration and Metastasis. Cancer Res..

[28] Gujral T.S., Chan M., Peshkin L., Sorger P.K., Kirschner M.W., MacBeath G. (2014). A noncanonical Frizzled2 pathway regulates epithelial-mesenchymal transition and metastasis. Cell.

[29] Kotrbová A., Ovesná P., Gybel T., Radaszkiewicz T., Bednaříková M., Hausnerová J., Jandáková E., Minář L., Crha I., Weinberger V. (2020). WNT signaling inducing activity in ascites predicts poor outcome in ovarian cancer. Theranostics.

[30] VanderVorst K., Dreyer C.A., Hatakeyama J., Bell G.R.R., Learn J.A., Berg A.L., Hernandez M., Lee H., Collins S.R., Carraway K.L. (2023). Vangl-dependent Wnt/planar cell polarity signaling mediates collective breast carcinoma motility and distant metastasis. Breast Cancer Res..

[31] Liu Y., Deng H., Liang L., Zhang G., Xia J., Ding K., Tang N., Wang K. (2021). Depletion of VPS35 attenuates metastasis of hepatocellular carcinoma by restraining the Wnt/PCP signaling pathway. Genes. Dis..

[32] Gong B., Shen W., Xiao W., Meng Y., Meng A., Jia S. (2017). The Sec14-like phosphatidylinositol transfer proteins Sec14l3/SEC14L2 act as GTPase proteins to mediate Wnt/Ca(2+) signaling. Elife.

[33] Ren R., Guo J., Chen Y., Zhang Y., Chen L., Xiong W. (2021). The role of Ca(2+)/Calcineurin/NFAT signalling pathway in osteoblastogenesis. Cell Prolif..

[34] Sarabia-Sánchez M.A., Moreno-Londoño A.P., Castañeda-Patlán M.C., Alvarado-Ortiz E., Martínez-Morales J.C., Robles-Flores M. (2023). Non-canonical Wnt/Ca^2+^ signaling is essential to promote self-renewal and proliferation in colon cancer stem cells. Front. Oncol..

[35] Sidaway P. (2015). Prostate cancer: Wnt signalling induces resistance. Nat. Rev. Urol..

[36] Routledge D., Scholpp S. (2019). Mechanisms of intercellular Wnt transport. Development.

[37] Zhang L., Wrana J.L. (2014). The emerging role of exosomes in Wnt secretion and transport. Curr. Opin. Genet. Dev..

[38] Masaki T., Shiratori Y., Rengifo W., Igarashi K., Yamagata M., Kurokohchi K., Uchida N., Miyauchi Y., Yoshiji H., Watanabe S. (2003). Cyclins and cyclin-dependent kinases: Comparative study of hepatocellular carcinoma versus cirrhosis. Hepatology.

[39] Zhou J., Shi K., Huang W., Zhang Y., Chen Q., Mou T., Wu Z., Wei X. (2023). LncRNA RPPH1 acts as a molecular sponge for miR-122 to regulate Wnt1/β-catenin signaling in hepatocellular carcinoma. Int. J. Med. Sci..

[40] Zhang X., Lu X., Shi J., Li Y., Li Y., Tao R., Huang L., Tang Y., Zhu X., Li M. (2024). Bufalin suppresses hepatocellular carcinogenesis by targeting M2 macrophage-governed Wnt1/β-catenin signaling. Phytomedicine.

[41] Jiang Y., Han Q., Zhao H., Zhang J. (2021). Promotion of epithelial-mesenchymal transformation by hepatocellular carcinoma-educated macrophages through Wnt2b/β-catenin/c-Myc signaling and reprogramming glycolysis. J. Exp. Clin. Cancer Res..

[42] Shimizu H., Julius M.A., Giarré M., Zheng Z., Brown A.M., Kitajewski J. (1997). Transformation by Wnt family proteins correlates with regulation of beta-catenin. Cell Growth Differ..

[43] Pan L., Yao M., Zheng W., Gu J., Yang X., Qiu L., Cai Y., Wu W., Yao D. (2016). Abnormality of Wnt3a expression as novel specific biomarker for diagnosis and differentiation of hepatocellular carcinoma. Tumour Biol..

[44] Zhang X.Z., Mo X.C., Wang Z.T., Sun R., Sun D.Q. (2024). N-glycosylation of Wnt3 regulates the progression of hepatocellular carcinoma by affecting Wnt/β-catenin signal pathway. World J. Gastrointest. Oncol..

[45] Lu C., He Y., Duan J., Yang Y., Zhong C., Zhang J., Liao W., Huang X., Zhu R., Li M. (2017). Expression of Wnt3a in hepatocellular carcinoma and its effects on cell cycle and metastasis. Int. J. Oncol..

[46] Zheng W., Yao M., Fang M., Pan L., Wang L., Yang J., Dong Z., Yao D. (2019). Oncogenic Wnt3a: A Candidate Specific Marker and Novel Molecular Target for Hepatocellular Carcinoma. J. Cancer.

[47] Nambotin S.B., Tomimaru Y., Merle P., Wands J.R., Kim M. (2012). Functional consequences of WNT3/Frizzled7-mediated signaling in non-transformed hepatic cells. Oncogenesis.

[48] Liu G., Jiang S., Wang C., Jiang W., Liu Z., Liu C., Saiyin H., Yang X., Shen S., Jiang D. (2012). Zinc finger transcription factor 191, directly binding to β-catenin promoter, promotes cell proliferation of hepatocellular carcinoma. Hepatology.

[49] Liu Y., Wu D., Cheng H., Chen L., Zhang W., Zou L., Gao Q., Zhao Z., Chen Q., Zeng W. (2021). Wnt8B, transcriptionally regulated by ZNF191, promotes cell proliferation of hepatocellular carcinoma via Wnt signaling. Cancer Sci..

[50] Wend P., Wend K., Krum S.A., Miranda-Carboni G.A. (2012). The role of WNT10B in physiology and disease. Acta Physiol..

[51] Zhang S., Zhang F., Chen Q., Wan C., Xiong J., Xu J. (2019). CRISPR/Cas9-mediated knockout of NSD1 suppresses the hepatocellular carcinoma development via the NSD1/H3/Wnt10b signaling pathway. J. Exp. Clin. Cancer Res..

[52] Li P., Cao Y., Li Y., Zhou L., Liu X., Geng M. (2014). Expression of Wnt-5a and β-catenin in primary hepatocellular carcinoma. Int. J. Clin. Exp. Pathol..

[53] Wang T., Liu X., Wang J. (2019). Up-regulation of Wnt5a inhibits proliferation and migration of hepatocellular carcinoma cells. J. Cancer Res. Ther..

[54] Geng M., Cao Y.C., Chen Y.J., Jiang H., Bi L.Q., Liu X.H. (2012). Loss of Wnt5a and Ror2 protein in hepatocellular carcinoma associated with poor prognosis. World J. Gastroenterol..

[55] Mikels A., Minami Y., Nusse R. (2009). Ror2 receptor requires tyrosine kinase activity to mediate Wnt5A signaling. J. Biol. Chem..

[56] Wakizaka K., Kamiyama T., Wakayama K., Orimo T., Shimada S., Nagatsu A., Kamachi H., Yokoo H., Fukai M., Kobayashi N. (2020). Role of Wnt5a in suppressing invasiveness of hepatocellular carcinoma via epithelial-mesenchymal transition. Oncol. Lett..

[57] Yuzugullu H., Benhaj K., Ozturk N., Senturk S., Celik E., Toylu A., Tasdemir N., Yilmaz M., Erdal E., Akcali K.C. (2009). Canonical Wnt signaling is antagonized by noncanonical Wnt5a in hepatocellular carcinoma cells. Mol. Cancer.

[58] Lan L., Wang W., Huang Y., Zhao C., Bu X. (2019). WNT7A Overexpression Inhibits Growth and Migration of Hepatocellular Carcinoma via the β-Catenin Independent Pathway. Biomed. Res. Int..

[59] Toyama T., Lee H.C., Koga H., Wands J.R., Kim M. (2010). Noncanonical Wnt11 inhibits hepatocellular carcinoma cell proliferation and migration. Mol. Cancer Res..

[60] Matsumoto S., Harada A., Seta M., Akita M., Gon H., Fukumoto T., Kikuchi A. (2023). Wnt Signaling Stimulates Cooperation between GREB1 and HNF4α to Promote Proliferation in Hepatocellular Carcinoma. Cancer Res..

[61] Planutis K., Planutiene M., Nguyen A.V., Moyer M.P., Holcombe R.F. (2013). Invasive colon cancer, but not non-invasive adenomas induce a gradient effect of Wnt pathway receptor frizzled 1 (Fz1) expression in the tumor microenvironment. J. Transl. Med..

[62] Ramadan A., Ghanem H.M., Mohamed A.A., Elshobaky M., El Agawy W., Gawad E., Eldeeb H.H., Ezz Al Arab M.R., Kamal M.M. (2023). GPC3 gene expression and allelic discrimination of FZD7 gene in Egyptian patients with hepatocellular carcinoma. Rep. Pr. Oncol. Radiother..

[63] Gu Y., Wang Z., Liang G., Peng J., Zhang X., Yu T., Ding C., Li Z. (2024). SIRT7 stabilizes β-catenin and promotes canonical Wnt activation via upregulating FZD7. Life Sci..

[64] Merle P., Kim M., Herrmann M., Gupte A., Lefrançois L., Califano S., Trépo C., Tanaka S., Vitvitski L., de la Monte S. (2005). Oncogenic role of the frizzled-7/beta-catenin pathway in hepatocellular carcinoma. J. Hepatol..

[65] Wang J., Yu H., Dong W., Zhang C., Hu M., Ma W., Jiang X., Li H., Yang P., Xiang D. (2023). N6-Methyladenosine-Mediated Up-Regulation of FZD10 Regulates Liver Cancer Stem Cells’ Properties and Lenvatinib Resistance Through WNT/β-Catenin and Hippo Signaling Pathways. Gastroenterology.

[66] Tung E.K., Wong B.Y., Yau T.O., Ng I.O. (2012). Upregulation of the Wnt co-receptor LRP6 promotes hepatocarcinogenesis and enhances cell invasion. PLoS ONE.

[67] Roslan Z., Muhamad M., Selvaratnam L., Ab-Rahim S. (2019). The Roles of Low-Density Lipoprotein Receptor-Related Proteins 5, 6, and 8 in Cancer: A Review. J. Oncol..

[68] Lee S., Lee M.J., Zhang J., Yu G.R., Kim D.G. (2016). C-terminal-truncated HBV X promotes hepato-oncogenesis through inhibition of tumor-suppressive β-catenin/BAMBI signaling. Exp. Mol. Med..

[69] Ou H., Chen Z., Xiang L., Fang Y., Xu Y., Liu Q., Hu Z., Li X., Huang Y., Yang D. (2019). Frizzled 2-induced epithelial-mesenchymal transition correlates with vasculogenic mimicry, stemness, and Hippo signaling in hepatocellular carcinoma. Cancer Sci..

[70] Asano T., Yamada S., Fuchs B.C., Takami H., Hayashi M., Sugimoto H., Fujii T., Tanabe K.K., Kodera Y. (2017). Clinical implication of Frizzled 2 expression and its association with epithelial-to-mesenchymal transition in hepatocellular carcinoma. Int. J. Oncol..

[71] Ren D., Minami Y., Nishita M. (2011). Critical role of Wnt5a-Ror2 signaling in motility and invasiveness of carcinoma cells following Snail-mediated epithelial-mesenchymal transition. Genes. Cells.

[72] Wakizaka K., Kamiyama T., Kakisaka T., Orimo T., Nagatsu A., Aiyama T., Shichi S., Taketomi A. (2024). Expression of Wnt5a and ROR2, Components of the Noncanonical Wnt-Signaling Pathway, is Associated with Tumor Differentiation in Hepatocellular Carcinoma. Ann. Surg. Oncol..

[73] Li L.Y., Yang J.F., Rong F., Luo Z.P., Hu S., Fang H., Wu Y., Yao R., Kong W.H., Feng X.W. (2021). ZEB1 serves an oncogenic role in the tumourigenesis of HCC by promoting cell proliferation, migration, and inhibiting apoptosis via Wnt/β-catenin signaling pathway. Acta Pharmacol. Sin..

[74] Lu C., Jia S., Zhao S., Shao X. (2019). MiR-342 regulates cell proliferation and apoptosis in hepatocellular carcinoma through Wnt/β-catenin signaling pathway. Cancer Biomark..

[75] Bai Z., Xia X., Lu J. (2020). MicroRNA-639 is Down-Regulated in Hepatocellular Carcinoma Tumor Tissue and Inhibits Proliferation and Migration of Human Hepatocellular Carcinoma Cells Through the KAT7/Wnt/β-Catenin Pathway. Med. Sci. Monit..

[76] Zhu J., Wu Y., Lao S., Shen J., Yu Y., Fang C., Zhang N., Li Y., Zhang R. (2021). Targeting TRIM54/Axin1/β-Catenin Axis Prohibits Proliferation and Metastasis in Hepatocellular Carcinoma. Front. Oncol..

[77] Li S., Wang J., Chen H., Hou J., Shen T., Li J., Zhou B., Zhang B., Liu H., Jiang D.K. (2023). TRIM16 E121D variant affects the risk and prognosis of hepatocellular carcinoma by modulating the Wnt/β-catenin pathway. Mol. Carcinog..

[78] Tong Q., Yi M., Kong P., Xu L., Huang W., Niu Y., Gan X., Zhan H., Tian R., Yan D. (2022). TRIM36 inhibits tumorigenesis through the Wnt/β-catenin pathway and promotes caspase-dependent apoptosis in hepatocellular carcinoma. Cancer Cell Int..

[79] Zanotti S., Canalis E. (2012). Nemo-like kinase inhibits osteoblastogenesis by suppressing bone morphogenetic protein and WNT canonical signaling. J. Cell Biochem..

[80] Liu Z., Zhong Y., Chen Y.J., Chen H. (2019). SOX11 regulates apoptosis and cell cycle in hepatocellular carcinoma via Wnt/β-catenin signaling pathway. Biotechnol. Appl. Biochem..

[81] Chen H., Ao Q., Wang Y., Qian Y., Cheng Q., Zhang W. (2024). SOX11 as a potential prognostic biomarker in hepatocellular carcinoma linked to immune infiltration and ferroptosis. Chin. J. Cancer Res..

[82] Wei B., Chen H., Chen X., Guo D., Hong L., Zheng S. (2022). Sox15 Methylation Inhibits Cell Proliferation Through Wnt Signaling in Hepatocellular Carcinoma. Front. Oncol..

[83] Del Prete A., Schioppa T., Tiberio L., Stabile H., Sozzani S. (2017). Leukocyte trafficking in tumor microenvironment. Curr. Opin. Pharmacol..

[84] Patel S., Alam A., Pant R., Chattopadhyay S. (2019). Wnt Signaling and Its Significance Within the Tumor Microenvironment: Novel Therapeutic Insights. Front. Immunol..

[85] Pandit H., Li Y., Li X., Zhang W., Li S., Martin R.C.G. (2018). Enrichment of cancer stem cells via β-catenin contributing to the tumorigenesis of hepatocellular carcinoma. BMC Cancer.

[86] Zhang X., Li N., Zhu Y., Wen W. (2022). The role of mesenchymal stem cells in the occurrence, development, and therapy of hepatocellular carcinoma. Cancer Med..

[87] Giannelli G., Koudelkova P., Dituri F., Mikulits W. (2016). Role of epithelial to mesenchymal transition in hepatocellular carcinoma. J. Hepatol..

[88] Qi Y., Wang H., Zhang Q., Liu Z., Wang T., Wu Z., Wu W. (2022). CAF-Released Exosomal miR-20a-5p Facilitates HCC Progression via the LIMA1-Mediated β-Catenin Pathway. Cells.

[89] Chen S., Shen J., Zhao J., Wang J., Shan T., Li J., Xu M., Chen X., Liu Y., Cao G. (2020). Magnolol Suppresses Pancreatic Cancer Development In Vivo and In Vitro via Negatively Regulating TGF-β/Smad Signaling. Front. Oncol..

[90] Creighton C.J., Gibbons D.L., Kurie J.M. (2013). The role of epithelial-mesenchymal transition programming in invasion and metastasis: A clinical perspective. Cancer Manag. Res..

[91] Zhai B., Yan H.X., Liu S.Q., Chen L., Wu M.C., Wang H.Y. (2008). Reduced expression of E-cadherin/catenin complex in hepatocellular carcinomas. World J. Gastroenterol..

[92] Guo F., Wang H., Jiang M., Yang Q., Xiang Q., Zhou H., Hu X., Hao K., Yang J., Cao H. (2020). TDP-43 induces EMT and promotes hepatocellular carcinoma metastasis via activating Wnt/β-catenin signaling pathway. Am. J. Cancer Res..

[93] Yang Y., Ye Y.C., Chen Y., Zhao J.L., Gao C.C., Han H., Liu W.C., Qin H.Y. (2018). Crosstalk between hepatic tumor cells and macrophages via Wnt/β-catenin signaling promotes M2-like macrophage polarization and reinforces tumor malignant behaviors. Cell Death Dis..

[94] Chen Y., Wen H., Zhou C., Su Q., Lin Y., Xie Y., Huang Y., Qiu Q., Lin J., Huang X. (2019). TNF-α derived from M2 tumor-associated macrophages promotes epithelial-mesenchymal transition and cancer stemness through the Wnt/β-catenin pathway in SMMC-7721 hepatocellular carcinoma cells. Exp. Cell Res..

[95] Xu W.D., Wang J., Yuan T.L., Li Y.H., Yang H., Liu Y., Zhao Y., Herrmann M. (2016). Interactions between canonical Wnt signaling pathway and MAPK pathway regulate differentiation, maturation and function of dendritic cells. Cell Immunol..

[96] Ruiz de Galarreta M., Bresnahan E., Molina-Sánchez P., Lindblad K.E., Maier B., Sia D., Puigvehi M., Miguela V., Casanova-Acebes M., Dhainaut M. (2019). β-Catenin Activation Promotes Immune Escape and Resistance to Anti-PD-1 Therapy in Hepatocellular Carcinoma. Cancer Discov..

[97] Xiao X., Mo H., Tu K. (2020). CTNNB1 mutation suppresses infiltration of immune cells in hepatocellular carcinoma through miRNA-mediated regulation of chemokine expression. Int. Immunopharmacol..

[98] Hu J., Dong A., Fernandez-Ruiz V., Shan J., Kawa M., Martínez-Ansó E., Prieto J., Qian C. (2009). Blockade of Wnt signaling inhibits angiogenesis and tumor growth in hepatocellular carcinoma. Cancer Res..

[99] Wang Q., Liang N., Yang T., Li Y., Li J., Huang Q., Wu C., Sun L., Zhou X., Cheng X. (2021). DNMT1-mediated methylation of BEX1 regulates stemness and tumorigenicity in liver cancer. J. Hepatol..

[100] Chang H.L., Bamodu O.A., Ong J.R., Lee W.H., Yeh C.T., Tsai J.T. (2020). Targeting the Epigenetic Non-Coding RNA MALAT1/Wnt Signaling Axis as a Therapeutic Approach to Suppress Stemness and Metastasis in Hepatocellular Carcinoma. Cells.

[101] Zhu G.Q., Wang Y., Wang B., Liu W.R., Dong S.S., Chen E.B., Cai J.L., Wan J.L., Du J.X., Song L.N. (2022). Targeting HNRNPM Inhibits Cancer Stemness and Enhances Antitumor Immunity in Wnt-activated Hepatocellular Carcinoma. Cell Mol. Gastroenterol. Hepatol..

[102] Leung H.W., Leung C.O.N., Lau E.Y., Chung K.P.S., Mok E.H., Lei M.M.L., Leung R.W.H., Tong M., Keng V.W., Ma C. (2021). EPHB2 Activates β-Catenin to Enhance Cancer Stem Cell Properties and Drive Sorafenib Resistance in Hepatocellular Carcinoma. Cancer Res..

[103] Levine B., Kroemer G. (2008). Autophagy in the pathogenesis of disease. Cell.

[104] White E. (2015). The role for autophagy in cancer. J. Clin. Investig..

[105] Glick D., Barth S., Macleod K.F. (2010). Autophagy: Cellular and molecular mechanisms. J. Pathol..

[106] Petherick K.J., Williams A.C., Lane J.D., Ordóñez-Morán P., Huelsken J., Collard T.J., Smartt H.J., Batson J., Malik K., Paraskeva C. (2013). Autolysosomal β-catenin degradation regulates Wnt-autophagy-p62 crosstalk. Embo J..

[107] Cui J., Shen H.M., Lim L.H.K. (2020). The Role of Autophagy in Liver Cancer: Crosstalk in Signaling Pathways and Potential Therapeutic Targets. Pharmaceuticals.

[108] Turcios L., Chacon E., Garcia C., Eman P., Cornea V., Jiang J., Spear B., Liu C., Watt D.S., Marti F. (2019). Autophagic flux modulation by Wnt/β-catenin pathway inhibition in hepatocellular carcinoma. PLoS ONE.

[109] Toshima T., Shirabe K., Matsumoto Y., Yoshiya S., Ikegami T., Yoshizumi T., Soejima Y., Ikeda T., Maehara Y. (2014). Autophagy enhances hepatocellular carcinoma progression by activation of mitochondrial β-oxidation. J. Gastroenterol..

[110] Davis B.A., Hogan E.M., Boron W.F. (1994). Shrinkage-induced activation of Na(+)-H+ exchange in barnacle muscle fibers. Am. J. Physiol..

[111] Fan Q., Yang L., Zhang X., Ma Y., Li Y., Dong L., Zong Z., Hua X., Su D., Li H. (2018). Autophagy promotes metastasis and glycolysis by upregulating MCT1 expression and Wnt/β-catenin signaling pathway activation in hepatocellular carcinoma cells. J. Exp. Clin. Cancer Res..

[112] Leung C.O.N., Yang Y., Leung R.W.H., So K.K.H., Guo H.J., Lei M.M.L., Muliawan G.K., Gao Y., Yu Q.Q., Yun J.P. (2023). Broad-spectrum kinome profiling identifies CDK6 upregulation as a driver of lenvatinib resistance in hepatocellular carcinoma. Nat. Commun..

[113] Xu J., Wan Z., Tang M., Lin Z., Jiang S., Ji L., Gorshkov K., Mao Q., Xia S., Cen D. (2020). N(6)-methyladenosine-modified CircRNA-SORE sustains sorafenib resistance in hepatocellular carcinoma by regulating β-catenin signaling. Mol. Cancer.

[114] Li T.T., Mou J., Pan Y.J., Huo F.C., Du W.Q., Liang J., Wang Y., Zhang L.S., Pei D.S. (2021). MicroRNA-138-1-3p sensitizes sorafenib to hepatocellular carcinoma by targeting PAK5 mediated β-catenin/ABCB1 signaling pathway. J. Biomed. Sci..

[115] Shi C.J., Lv M.Y., Deng L.Q., Zeng W.Q., Fu W.M., Zhang J.F. (2023). Linc-ROR drive adriamycin resistance by targeting AP-2α/Wnt/β-catenin axis in hepatocellular carcinoma. Cell Biol. Toxicol..

[116] Fang L., Gao C., Bai R.X., Wang H.F., Du S.Y. (2021). Overexpressed sFRP3 exerts an inhibitory effect on hepatocellular carcinoma via inactivation of the Wnt/β-catenin signaling pathway. Cancer Gene Ther..

[117] Lin X.H., Liu H.H., Hsu S.J., Zhang R., Chen J., Chen J., Gao D.M., Cui J.F., Ren Z.G., Chen R.X. (2020). Norepinephrine-stimulated HSCs secrete sFRP1 to promote HCC progression following chronic stress via augmentation of a Wnt16B/β-catenin positive feedback loop. J. Exp. Clin. Cancer Res..

[118] Kim M.J., Huang Y., Park J.I. (2020). Targeting Wnt Signaling for Gastrointestinal Cancer Therapy: Present and Evolving Views. Cancers.

[119] Yu F., Yu C., Li F., Zuo Y., Wang Y., Yao L., Wu C., Wang C., Ye L. (2021). Wnt/β-catenin signaling in cancers and targeted therapies. Signal Transduct. Target. Ther..

[120] Wei W., Chua M.S., Grepper S., So S.K. (2011). Soluble Frizzled-7 receptor inhibits Wnt signaling and sensitizes hepatocellular carcinoma cells towards doxorubicin. Mol. Cancer.

[121] Suda T., Yamashita T., Sunagozaka H., Okada H., Nio K., Sakai Y., Yamashita T., Mizukoshi E., Honda M., Kaneko S. (2022). Dickkopf-1 Promotes Angiogenesis and is a Biomarker for Hepatic Stem Cell-like Hepatocellular Carcinoma. Int. J. Mol. Sci..

[122] Tao Y.M., Liu Z., Liu H.L. (2013). Dickkopf-1 (DKK1) promotes invasion and metastasis of hepatocellular carcinoma. Dig. Liver Dis..

[123] Haas M.S., Kagey M.H., Heath H., Schuerpf F., Rottman J.B., Newman W. (2021). mDKN-01, a Novel Anti-DKK1 mAb, Enhances Innate Immune Responses in the Tumor Microenvironment. Mol. Cancer Res..

[124] Xiao J., Xing F., Liu Y., Lv Y., Wang X., Ling M.T., Gao H., Ouyang S., Yang M., Zhu J. (2018). Garlic-derived compound S-allylmercaptocysteine inhibits hepatocarcinogenesis through targeting LRP6/Wnt pathway. Acta Pharm. Sin. B.

[125] Ma L., Wang X., Jia T., Wei W., Chua M.S., So S. (2015). Tankyrase inhibitors attenuate WNT/β-catenin signaling and inhibit growth of hepatocellular carcinoma cells. Oncotarget.

[126] Huang J., Qu Q., Guo Y., Xiang Y., Feng D. (2020). Tankyrases/β-catenin Signaling Pathway as an Anti-proliferation and Anti-metastatic Target in Hepatocarcinoma Cell Lines. J. Cancer.

[127] Thorne C.A., Hanson A.J., Schneider J., Tahinci E., Orton D., Cselenyi C.S., Jernigan K.K., Meyers K.C., Hang B.I., Waterson A.G. (2010). Small-molecule inhibition of Wnt signaling through activation of casein kinase 1α. Nat. Chem. Biol..

[128] Ho S.Y., Keller T.H. (2015). The use of porcupine inhibitors to target Wnt-driven cancers. Bioorg Med. Chem. Lett..

[129] Liu J., Pan S., Hsieh M.H., Ng N., Sun F., Wang T., Kasibhatla S., Schuller A.G., Li A.G., Cheng D. (2013). Targeting Wnt-driven cancer through the inhibition of Porcupine by LGK974. Proc. Natl. Acad. Sci. USA.

[130] Shah K., Panchal S., Patel B. (2021). Porcupine inhibitors: Novel and emerging anti-cancer therapeutics targeting the Wnt signaling pathway. Pharmacol. Res..

[131] Fako V., Yu Z., Henrich C.J., Ransom T., Budhu A.S., Wang X.W. (2016). Inhibition of wnt/β-catenin Signaling in Hepatocellular Carcinoma by an Antipsychotic Drug Pimozide. Int. J. Biol. Sci..

[132] Kimura K., Ikoma A., Shibakawa M., Shimoda S., Harada K., Saio M., Imamura J., Osawa Y., Kimura M., Nishikawa K. (2017). Safety, Tolerability, and Preliminary Efficacy of the Anti-Fibrotic Small Molecule PRI-724, a CBP/β-Catenin Inhibitor, in Patients with Hepatitis C Virus-related Cirrhosis: A Single-Center, Open-Label, Dose Escalation Phase 1 Trial. EBioMedicine.

[133] Gabata R., Harada K., Mizutani Y., Ouchi H., Yoshimura K., Sato Y., Kitao A., Kimura K., Kouji H., Miyashita T. (2020). Anti-tumor Activity of the Small Molecule Inhibitor PRI-724 Against β-Catenin-activated Hepatocellular Carcinoma. Anticancer. Res..

[134] Feng H., Zhu M., Zhang R., Wang Q., Li W., Dong X., Chen Y., Lu Y., Liu K., Lin B. (2019). GATA5 inhibits hepatocellular carcinoma cells malignant behaviours by blocking expression of reprogramming genes. J. Cell Mol. Med..

[135] Gedaly R., Galuppo R., Daily M.F., Shah M., Maynard E., Chen C., Zhang X., Esser K.A., Cohen D.A., Evers B.M. (2014). Targeting the Wnt/β-catenin signaling pathway in liver cancer stem cells and hepatocellular carcinoma cell lines with FH535. PLoS ONE.

[136] Vilchez V., Turcios L., Marti F., Gedaly R. (2016). Targeting Wnt/β-catenin pathway in hepatocellular carcinoma treatment. World J. Gastroenterol..

[137] Wang Q.Y., Feng Y.J., Ji R. (2020). High expression of WISP1 promotes metastasis and predicts poor prognosis in hepatocellular carcinoma. Eur. Rev. Med. Pharmacol. Sci..

[138] Gao H., Yin F.F., Guan D.X., Feng Y.X., Zheng Q.W., Wang X., Zhu M., Zhang X.L., Cheng S.Q., Chen T.W. (2019). Liver cancer: WISP3 suppresses hepatocellular carcinoma progression by negative regulation of β-catenin/TCF/LEF signalling. Cell Prolif..

[139] Zhu C., Luo X., Wu J., Liu Y., Liu L., Ma S., Xie R., Wang S., Ji W. (2021). TM4SF1, a binding protein of DVL2 in hepatocellular carcinoma, positively regulates beta-catenin/TCF signalling. J. Cell Mol. Med..

